# Muscle-Specific Splicing Factors ASD-2 and SUP-12 Cooperatively Switch Alternative Pre-mRNA Processing Patterns of the ADF/Cofilin Gene in *Caenorhabditis elegans*

**DOI:** 10.1371/journal.pgen.1002991

**Published:** 2012-10-11

**Authors:** Genta Ohno, Kanako Ono, Marina Togo, Yohei Watanabe, Shoichiro Ono, Masatoshi Hagiwara, Hidehito Kuroyanagi

**Affiliations:** 1 Laboratory of Gene Expression, Graduate School of Biomedical Science, Tokyo Medical and Dental University, Tokyo, Japan; 2 Department of Functional Genomics, Medical Research Institute, Tokyo Medical and Dental University, Tokyo, Japan; 3 Research Fellowship for Young Scientists, Japan Society for the Promotion of Science (JSPS), Tokyo, Japan; 4 Department of Pathology, Emory University, Atlanta, Georgia, United States of America; 5 Graduate School of Medicine, Kyoto University, Kyoto, Japan; 6 Precursory Research for Embryonic Science and Technology (PRESTO), Japan Science and Technology Agency (JST), Kawaguchi, Saitama, Japan; University of California Los Angeles, United States of America

## Abstract

Pre–mRNAs are often processed in complex patterns in tissue-specific manners to produce a variety of protein isoforms from single genes. However, mechanisms orchestrating the processing of the entire transcript are not well understood. Muscle-specific alternative pre–mRNA processing of the *unc-60* gene in *Caenorhabditis elegans*, encoding two tissue-specific isoforms of ADF/cofilin with distinct biochemical properties in regulating actin organization, provides an excellent *in vivo* model of complex and tissue-specific pre–mRNA processing; it consists of a single first exon and two separate series of downstream exons. Here we visualize the complex muscle-specific processing pattern of the *unc-60* pre–mRNA with asymmetric fluorescence reporter minigenes. By disrupting juxtaposed CUAAC repeats and UGUGUG stretch in intron 1A, we demonstrate that these elements are required for retaining intron 1A, as well as for switching the processing patterns of the entire pre–mRNA from non-muscle-type to muscle-type. Mutations in genes encoding muscle-specific RNA–binding proteins ASD-2 and SUP-12 turned the colour of the *unc-60* reporter worms. ASD-2 and SUP-12 proteins specifically and cooperatively bind to CUAAC repeats and UGUGUG stretch in intron 1A, respectively, to form a ternary complex *in vitro*. Immunohistochemical staining and RT–PCR analyses demonstrate that ASD-2 and SUP-12 are also required for switching the processing patterns of the endogenous *unc-60* pre-mRNA from UNC-60A to UNC-60B in muscles. Furthermore, systematic analyses of partially spliced RNAs reveal the actual orders of intron removal for distinct mRNA isoforms. Taken together, our results demonstrate that muscle-specific splicing factors ASD-2 and SUP-12 cooperatively promote muscle-specific processing of the *unc-60* gene, and provide insight into the mechanisms of complex pre-mRNA processing; combinatorial regulation of a single splice site by two tissue-specific splicing regulators determines the binary fate of the entire transcript.

## Author Summary

Muscle is a specialized organ with specialized contractile apparatuses. A number of genes encoding contractile apparatus-related proteins undergo muscle-specific pre–mRNA processing. However, the molecular mechanisms and consequences of muscle-specific alternative pre–mRNA processing remain largely unknown. In this study, we reveal regulation mechanisms of pre–mRNA processing of the *unc-60* gene locus, encoding two tissue-specific isoforms of ADF/cofilin in *C. elegans*. The *unc-60A* and *unc-60B* genes share only the first exon, and UNC-60B protein is specifically expressed in muscle. We visualize the tissue-specific processing patterns of the *unc-60* pre–mRNA with green and red fluorescent proteins in living worms. We provide genetic, biochemical, and immunohistochemical evidence that muscle-specific RNA–binding proteins ASD-2 and SUP-12 cooperatively bind to specific motifs in intron 1A to retain intron 1A, which leads to skipping of exon 2A through 5A and splicing between exon 1 and 2B. Consistently, disruption of the splicing factors leads to expression of UNC-60A in muscle and suppresses paralysis of an *unc-60B*-specific mutant. Our study raises a model of step-by-step execution of complex co-transcriptional pre–mRNA processing and provides insight into the fate decision of the entire transcript.

## Introduction

Alternative pre-mRNA processing is a major way to produce a number of different mRNAs and proteins from one gene [1], [2]. Recent transcriptome analyses by deep sequencing estimated that more than 90% of human multi-exon genes undergo alternative processing and most alternative processing events are regulated in tissue-specific manners [3], [4]. These alternative pre-mRNA processing events are classified into seven elementary events: cassette exons, mutually exclusive exons, alternative 5′ splice sites, alternative 3′ splice sites, intron retention, alternative first exons and alternative polyadenylation sites [5], [6]. A variety of tissue-specific splicing factors and RNA secondary structures have been shown to regulate these elementary events in the minigene context or by knockdown and/or knockout experiments [7], [8], [9]. However, pre-mRNA processing in multicellular organisms is often complex due to various combinations of the elementary events and the molecular mechanisms by which tissue-specific factors regulate such complex alternative processing of the entire gene *in vivo* remain to be elucidated.

Muscle is one of tissues in which many genes undergo tissue-specific pre-mRNA processing [3], [4]. A number of muscle-specific protein isoforms are expressed by alternative pre-mRNA splicing and play adapted roles depending on the specific properties of muscle fiber types [10], [11], [12]. For instance, tissue-specific splicing generates functionally distinct isoforms of tropomyosin [13] and troponin T [14]. Global analyses of splicing patterns during development of heart and skeletal muscle revealed that splicing transitions of these genes occur at specific times [15], [16]. Bioinformatics analyses have revealed candidate *cis*-elements regulating muscle-specific splicing patterns [16], [17], [18]. In addition, several *trans*-acting splicing factors are known to regulate muscle-specific alternative splicing. These include muscleblind-like (MBNL) [19], RBFOX family [20], CUGBP and ETR-3 like factor (CELF) family [21], polypyrimidine tract binding protein (PTB) [22] and hnRNP H [23]. However, how multiple splicing factors coordinate regulation of specific splicing events is poorly understood.

**Figure 1:**
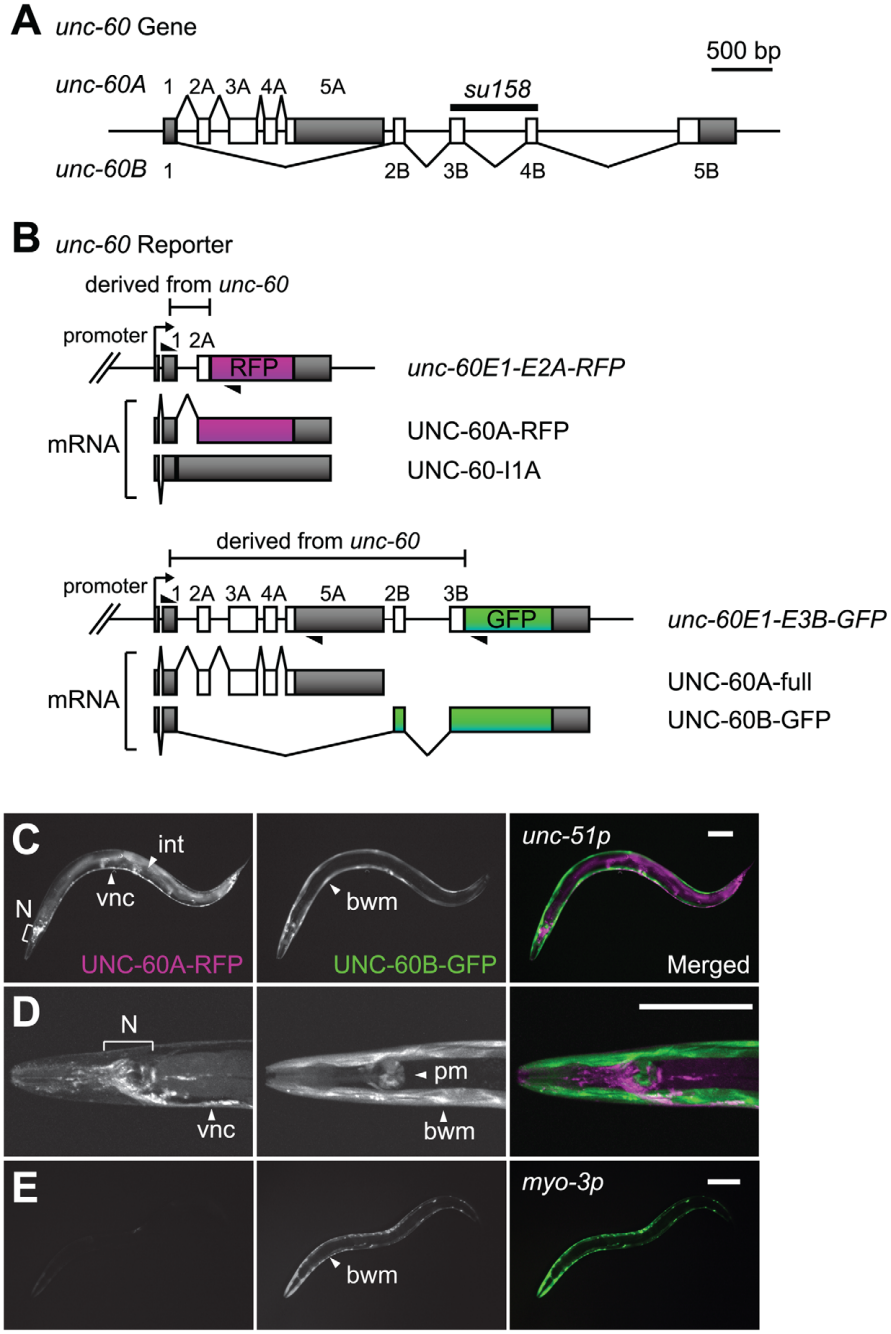
Visualization of tissue-specific alternative processing patterns of the *unc-60* transcript. (*A*) Schematic structure of the *unc-60* gene. Numbered boxes indicate exons. Predicted open reading frames (ORFs) are coloured in white and untranslated regions (UTRs) are in gray. The deleted region in *unc-60 (su158)* is indicated. (*B*) Schematic illustration of a pair of *unc-60* reporter minigenes, *unc-60E1-E2A-RFP* and *unc-60E1-E3B-GFP*, and UNC-60A- and UNC-60B-type mRNAs derived from them. cDNA cassettes and predicted ORFs for RFP and GFP are coloured in magenta and green, respectively. Triangles indicate positions and directions of primers used to check splicing patterns of mRNAs derived from the minigenes by RT-PCR. (*C* and *D*) Confocal images of transgenic *unc-60* reporter worms *ybEx1812 [unc-51::unc-60E1-E2A-RFP unc-51::unc-60E1-E3B-GFP]*. UNC-60A-RFP (left), UNC-60B-GFP (middle) and merged images (right) of an adult worm (*C*) and a head region at higher magnification (*D*). Anterior is to the left and dorsal is to the top. bwm, body wall muscles; int, intestine; N, neurons in head ganglia; pm, pharyngeal muscles; vnc, ventral nerve cord. Scale bars, 50 µm. (*E*) Confocal images of a transgenic *unc-60* reporter worm *ybIs1831 [myo-3::unc-60E1-E2A-RFP myo-3::unc-60E1-E3B-GFP]* shown as in (*C*) and (*D*).

Alternative processing of the *uncoordinated* (*unc*)*-60* gene in *Caenorhabditis elegans* provides an excellent model of muscle-specific and complex pre-mRNA processing of genes related to contractile apparatuses. The *unc-60* gene encodes two homologous proteins, UNC-60A and UNC-60B [24], which are members of the actin depolymerising factor (ADF)/cofilin family of actin-binding proteins that promote rapid turnover of the actin cytoskeleton [25]. The *unc-60* gene consists of a common first exon and two separate series of downstream exons, exons 2A through 5A for UNC-60A and exons 2B through 5B for UNC-60B (Figure 1A). Alternative choices of exons 2A–5A or exons 2B–5B result in tissue-specific expression patterns of the two ADF/cofilin isoforms: UNC-60A protein is expressed in most embryonic cells throughout embryogenesis and predominantly expressed in non-muscle tissues, while UNC-60B protein is mainly detected in body wall muscles [26]. Our biochemical and genetic studies demonstrated that the UNC-60 isoforms have distinct biochemical properties in the regulation of actin dynamics [27], [28] and different *in vivo* functions during development and in muscle organization [26], [29].

The structure of the *unc-60* gene and its expression patterns raise a question as to how the first exon and the two series of downstream exons are properly spliced in a tissue-specific manner. We previously reported genetic evidence that an RNA-binding protein SUP-12, which has only one RNA-recognition motif (RRM), is required for generation of muscle-specific UNC-60B mRNA [30]. However, the molecular mechanism by which SUP-12 regulates the muscle-specific alternative processing of the *unc-60* gene remains unclear. In this study, we applied a transgenic alternative splicing reporter system [31], [32], [33] to visualize muscle-specific alternative processing patterns of the *unc-60* pre-mRNA. We demonstrate that repression of excision of the intron between exon 1 and exon 2A is the fate-determining event for the *unc-60* transcript. We provide genetic and biochemical evidence that SUP-12 and another muscle-specific splicing regulator Alternative-Splicing-Defective-2 (ASD-2), a member of the signal transduction and activation of RNA (STAR) family of RNA-binding proteins [34], cooperatively repress excision of the first intron through specific binding to the intron. Our data provide *in vivo* evidence that combinatorial regulation of a single splice site by two tissue-specific splicing regulators determine the binary fate of the entire transcript that can potentially be processed into two alternative isoforms.

## Results

### Visualization of the muscle-specific alternative pre–mRNA processing of the *unc-60* gene

In order to visualize the binary processing patterns of the *unc-60* transcript *in vivo*, we intended to construct a pair of fluorescence alternative processing reporter minigenes. If the intron between exon 1 and exon 2A (hereafter called intron 1A) is excised prior to selection of exon 2B, it would be impossible to produce UNC-60B mRNA. We therefore assumed that excision of intron 1A should be repressed until exon 2B is transcribed in tissues where UNC-60B is expressed. On the basis of the assumption, we constructed an asymmetric pair of reporter minigenes, *unc-60E1-E2A-RFP* and *unc-60E1-E3B-GFP*. The *unc-60E1-E2A-RFP* cassette, carrying *unc-60* genomic DNA fragment from exon 1 through exon 2A (Figure 1B, top panel), was designed to monitor excision of intron 1A via expression of RFP-fusion protein (UNC-60A-RFP). If intron 1A is retained (UNC-60-I1A), RFP would not be expressed due to a premature termination codon in intron 1A (Figure 1B, top panel). On the other hand, the *unc-60E1-E3B-GFP* cassette, carrying *unc-60* genomic DNA fragment from exon 1 through exon 3B (Figure 1B, bottom panel), was designed to monitor UNC-60B-type processing via expression of GFP-fusion protein (UNC-60B-GFP). An intact UNC-60A isoform (UNC-60A-full) would be expressed in tissues where UNC-60A is expressed (Figure 1B, bottom panel).

We successfully visualized the alternative expression of the UNC-60 isoforms with the *unc-60* reporter cassettes under the control of the *unc-51* promoter that directs expression in a broad variety of tissues [35], [36]. As expected, the expression patterns of UNC-60A-RFP and UNC-60B-GFP varied between muscle and non-muscle tissues (Figure 1C, 1D). Non-muscle tissues including the nervous system and intestine expressed UNC-60A-RFP (Figure 1C, 1D, left panels), and muscle tissues such as body wall muscles and pharyngeal muscles expressed UNC-60B-GFP (Figure 1C, 1D, right panels). This result is consistent with our previous immunohistochemical studies showing that UNC-60A and UNC-60B proteins were detected in non-muscle and muscle tissues, respectively [26], [37]. We checked splicing patterns of mRNAs derived from the *unc-60* reporter cassettes by cloning and sequencing reverse transcription-polymerase chain reaction (RT-PCR) products, and confirmed that the four mRNA isoforms schematically shown in Figure 1B were actually generated in the transgenic worms (data not shown).

To focus on the muscle-specific control of the *unc-60* processing, we utilized *myo-3* promoter to drive expression of the *unc-60* reporter specifically in body wall muscles. Transgenic worms with an integrated transgene allele *ybIs1831 [myo-3::unc-60E1-E2A-RFP myo-3::unc-60E1-E3B-GFP]* predominantly expressed UNC-60B-GFP in body wall muscles (Figure 1E), consistent with the *unc-60* reporter expression in muscles (Figure 1C, 1D). We therefore used the *myo-3* promoter for further analyses described below.

### SUP-12 and another muscle-specific splicing factor ASD-2 regulate muscle-specific processing of the *unc-60* reporter

**Figure 2:**
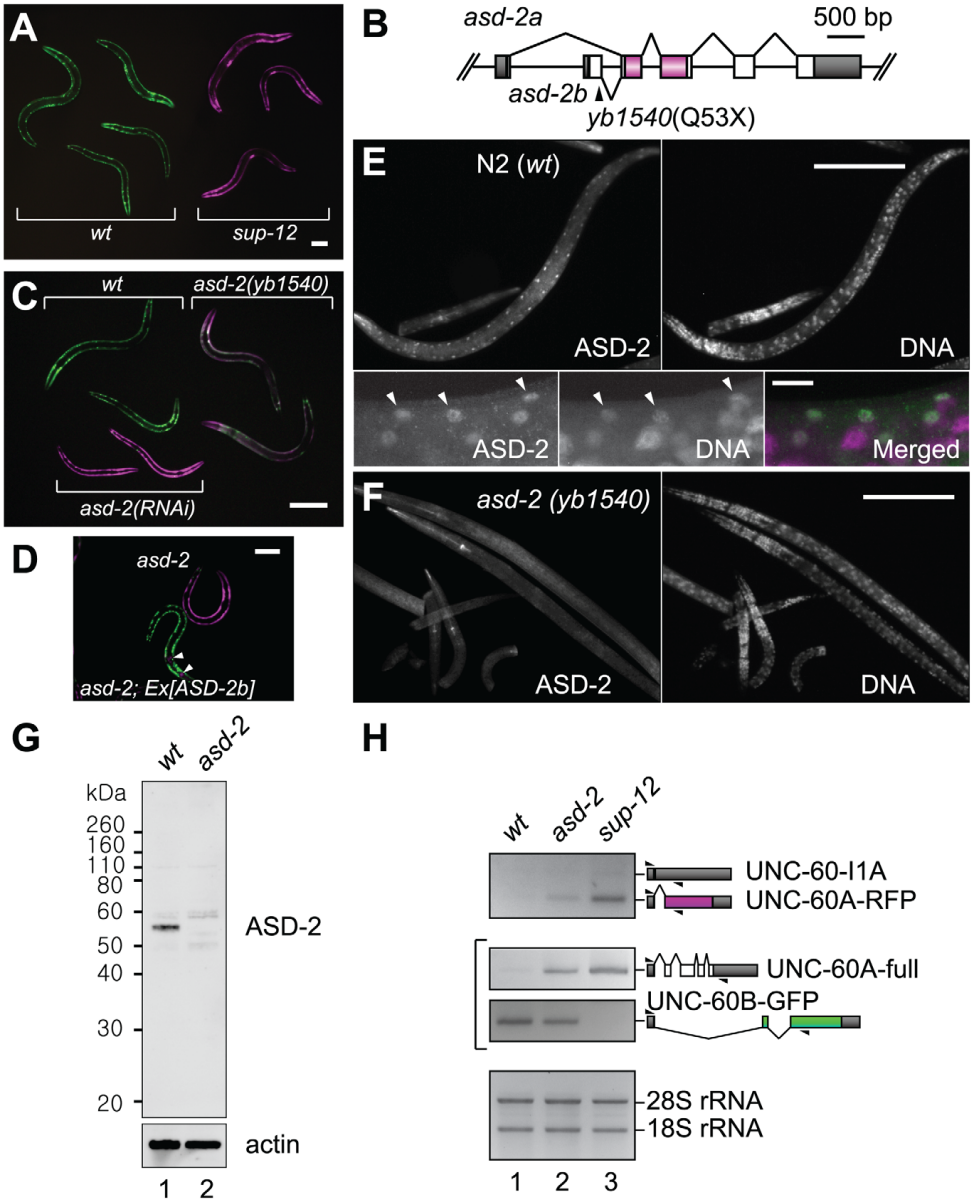
ASD-2 and SUP-12 regulate muscle-specific processing of the *unc-60* reporter in body wall muscles. (*A*) A micrograph of the *unc-60* reporter worms carrying the integrated allele *ybIs1831* in the wild-type (left) and *sup-12 (yb1253)* (right) backgrounds. (*B*) Schematic structure of the *asd-2* gene. The position of a nonsense mutation in *yb1540* is indicated. A region encoding STAR domain is coloured. (*C*) A micrograph of wild-type, *asd-2(yb1540)* mutant and *asd-2(RNAi)* worms carrying *ybIs1831*. (*D*) A micrograph of *asd-2(yb1540)* and *asd-2(yb1540); ybEx2266 [myo-2::mRFP myo-3::ASD-2b]* worms carrying *ybIs1831*. Arrowheads indicate RFP expression in pharynx as a marker for transgenesis. Scale bars in (*A*), (*C*) and (*D*), 50 µm. (*E*, *F*) Microphotographs of N2 (*E*) and *asd-2(yb1540)* (*F*) worms stained with anti-ASD-2b (ASD-2) and Hoechst 33258 (DNA). High-magnification and merged images are also indicated for N2 in bottom panels of (*E*). Arrowheads indicate nuclei of some of body wall muscle cells. Scale bars in (*E*) top panels and (*F*), 100 µm; in (*E*) bottom panels, 10 µm. (*G*) Western blotting with anti-ASD-2b. Lysates from synchronized L1 larvae of N2 (lane 1) and *asd-2(yb1540)* mutant (lane 2) were subjected to Western blotting with anti-ASD-2b (top) and anti-actin (bottom). (*H*) RT-PCR analysis of mRNAs derived from *ybIs1831* in the wild-type (lane 1), *asd-2 (yb1540)* (lane 2) and *sup-12 (yb1253)* (lane 3) backgrounds. RT-PCR products derived from *unc-60E1-E2A-RFP* (top) and *unc-60E1-E3B-GFP* (middle) and total RNAs (bottom) are shown. Splicing patterns of the mRNAs are schematically shown on the right. Triangles indicate positions and directions of the primers.

To test whether muscle-specific repression of UNC-60A-RFP and expression of UNC-60B-GFP from the *unc-60* reporter are similarly regulated by a muscle-specific splicing regulator SUP-12 to the endogenous mRNAs for UNC-60A and UNC-60B isoforms [30], we crossed the reporter allele *ybIs1831* with a presumptive null allele *sup-12 (yb1253)* [38]. As expected, the reporter worms clearly turned the colour from Green to Red in the *sup-12* background (Figure 2A), confirming that SUP-12 is required for the muscle-specific expression profile of the *unc-60* reporter.

In a previous study, we identified SUP-12 as a co-regulator of mutually exclusive exons of a fibroblast growth factor receptor gene *egg-laying-defective (egl)-15* [38]. In the case of repression of *egl-15* exon 5B, SUP-12 functions as a muscle-specific partner of the Fox-1 family proteins ASD-1 and FOX-1 [31], [38]. We therefore speculated that other regulator(s) may also be involved in the muscle-specific regulation of *unc-60*. As direct interaction between SUP-12 and ASD-1 in a yeast two-hybrid system had been reported in a worm interactome study [39], we screened for a putative co-regulator of the *unc-60* reporter by knocking down genes encoding possible SUP-12-interactors ASD-1, ASD-2, ETR-1, MEC-8, R02F2.5 and W02A11.3, deposited in the database (http://interactome.dfci.harvard.edu/). We performed RNA interference (RNAi) by feeding the reporter worms with bacterial clones targeting the six genes, and found that knockdown of *asd-2* led to expression of UNC-60A-RFP (Figure S1).

We previously identified ASD-2, an RNA-binding protein belonging to the STAR family, as a regulator of muscle-specific and developmentally regulated alternative splicing of a collagen gene *let-2* [32], [33]. The *asd-2* gene has alternative first exons and a non-lethal allele *asd-2 (yb1540)* has a nonsense mutation in the *asd-2b*-specific first exon (Figure 2B), which is used in body wall muscles and pharyngeal muscles [32]. The *unc-60* reporter worms exhibited weak Red phenotype in the *asd-2 (yb1540)* background (Figure 2C) and body wall muscle-specific expression of ASD-2b cDNA rescued the colour phenotype (Figure 2D), confirming that *asd-2b* is involved in the muscle-specific regulation of the *unc-60* reporter. To investigate subcellular localization of ASD-2, we raised polyclonal antibodies against recombinant full-length ASD-2b protein and stained wild-type and *asd-2 (yb1540)* worms with a purified immunoglobulin G (IgG) fraction (Figure 2E, 2F). Nuclei of body wall muscles, which are aligned along the dorsal and ventral periphery, are stained in the wild type (Figure 2E) and not in *asd-2* mutant (Figure 2F). In Western blotting, the same antibody detected a major band with an apparent molecular weight of 56 kDa in wild-type and not in *asd-2 (yb1540)* lysate (Figure 2G). These results indicated that ASD-2b is the major isoform and is predominantly localized in the nuclei of body wall muscles. RNAi by micro-injecting double-stranded RNA (dsRNA), a more effective method than feeding dsRNA-expressing bacteria, led to a stronger Red phenotype (Figure 2C), suggesting trace remaining activity of ASD-2 in *asd-2 (yb1540)* mutant.

To confirm splicing patterns of mRNAs derived from the *unc-60* reporter minigenes in body wall muscles, we performed RT-PCR analysis with minigene-specific primer sets (Figure 2H). In the wild-type background, UNC-60B-type mRNA, UNC-60B-GFP, was predominantly generated from *unc-60E1-E3B-GFP* (Figure 2H, middle panel, lane 1). A transcript derived from *unc-60E1-E2A-RFP* was almost undetectable (Figure 2H, top panel, lane 1), presumably due to rapid degradation of a non-productive mRNA isoform, UNC-60-I1A, by nonsense-mediated mRNA decay (NMD) [40]. On the other hand, the amount of UNC-60B-GFP was reduced and UNC-60A-type mRNAs, UNC60A-RFP and UNC-60A-full, were detected in *asd-2* and *sup-12* mutants (Figure 2H, lanes 2 and 3), consistent with their colour phenotypes shown in Figure 2C and 2A, respectively. These results confirmed that both SUP-12 and ASD-2 are responsible for switching the processing patterns of the *unc-60* reporter from UNC-60A-type to UNC-60B-type in body wall muscles.

### CUAAC repeats and UGUGUG stretch in intron 1A control the muscle-specific alternative processing of the *unc-60* reporter

**Figure 3:**
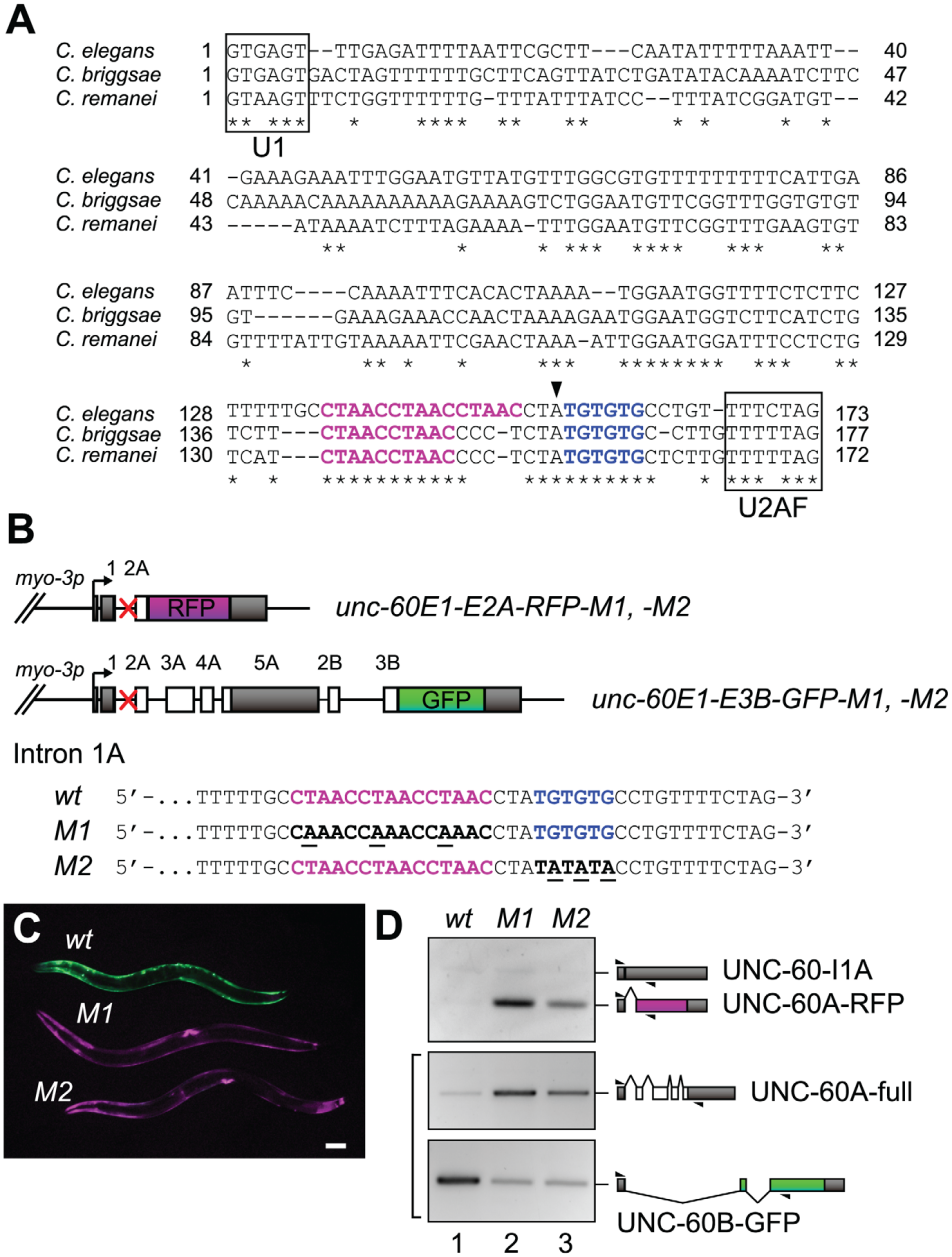
CUAAC repeats and UGUGUG stretch are required for muscle-specific alternative processing of the *unc-60* reporter. (*A*) Nucleotide sequence alignment of *unc-60* intron 1A from *C. elegans*, *C. briggsae* and *C. remanei*. Asterisks denote residues conserved among three species. CTAAC repeats and TGTGTG stretch are coloured in magenta and blue, respectively. Binding regions for U1 snRNP (U1) and U2 snRNP auxiliary factor (U2AF) are boxed. Arrowhead indicates a putative branch site. (*B*) Schematic illustrations of mutated pairs of *unc-60* reporter minigenes, *-M1* and *-M2* (top), and nucleotide sequences of the modified regions (bottom). Red crosses indicate positions of modification. Mutated residues in the mutant minigenes are underlined. CTAAC repeats and TGTGTG stretch are coloured as in (*A*). (*C*) A micrograph of transgenic worms expressing wild-type (top), *M1* (middle) and *M2* (bottom) pairs of the *unc-60* reporter minigenes. Anterior is to the left. Scale bar, 50 µm. (*D*) RT-PCR analysis of mRNAs derived from wild-type (lane 1), *M1* (lane 2) and *M2* (lane 3) pairs of *myo-3p-unc-60E1-E2A-RFP* (top) and *myo-3p-unc-60E1-E3B-GFP* (bottom). Schematic structures of the mRNAs are indicated on the right.

The experiments described above indicate that each of the *unc-60* reporter minigenes, even the shorter one, carries sufficient regulatory elements for ASD-2 and SUP-12 to switch from non-muscle-type to muscle-type processing. As regulatory elements for alternative splicing are often evolutionarily conserved in introns among nematodes [31], [32], [38], [41], we searched for conserved stretches in *unc-60* intron 1A in the *Caenorhabditis* genus. Alignment of nucleotide sequences available in WormBase (http://www.wormbase.org/) revealed that CTAAC repeats and TGTGTG stretch are highly conserved just upstream of the splice acceptor site (Figure 3A). To evaluate the roles of these elements in the muscle-specific processing of the *unc-60* reporter, we constructed two pairs of modified *unc-60* reporter minigenes *M1* and *M2*. In the *M1* pair, CTAAC repeats were mutagenized to CAAAC (Figure 3B). In the *M2* pair, TGTGTG were mutagenized to TATATA (Figure 3B). Disruption of either of the two elements resulted in Red phenotype (Figure 3C), phenocopying *sup-12* mutant (Figure 2A) and *asd-2 (RNAi)* worms (Figure 2C). RT-PCR analysis of mRNAs derived from the mutant reporters revealed that both *M1* and *M2* mutations increased production of UNC-60A-RFP (Figure 3D, top panel) and decreased expression of UNC-60B-GFP (Figure 3D, bottom panel), consistent with their colour phenotypes. These results confirmed that the colour phenotypes observed with the mutant reporters are due to altered patterns of pre-mRNA processing. We concluded that both CUAAC repeats and UGUGUG stretch are required for muscle-specific repression of intron 1A excision. Notably, expression of UNC-60A-full mRNA from the *M1* and *M2* mutants of *unc-60E1-E3B-GFP* minigene increased compared to the wild-type minigene (Figure 3D), indicating that the repression of intron 1A excision via CUAAC repeats and UGUGUG stretch is a crucial event to switch the processing patterns of the entire *unc-60E1-E3B-GFP* minigene from UNC-60A-type to UNC-60B-type.

### ASD-2 and SUP-12 cooperatively bind to *unc-60* intron 1A *in vitro* via direct and specific binding to CUAAC repeats and UGUGUG stretch, respectively

**Figure 4:**
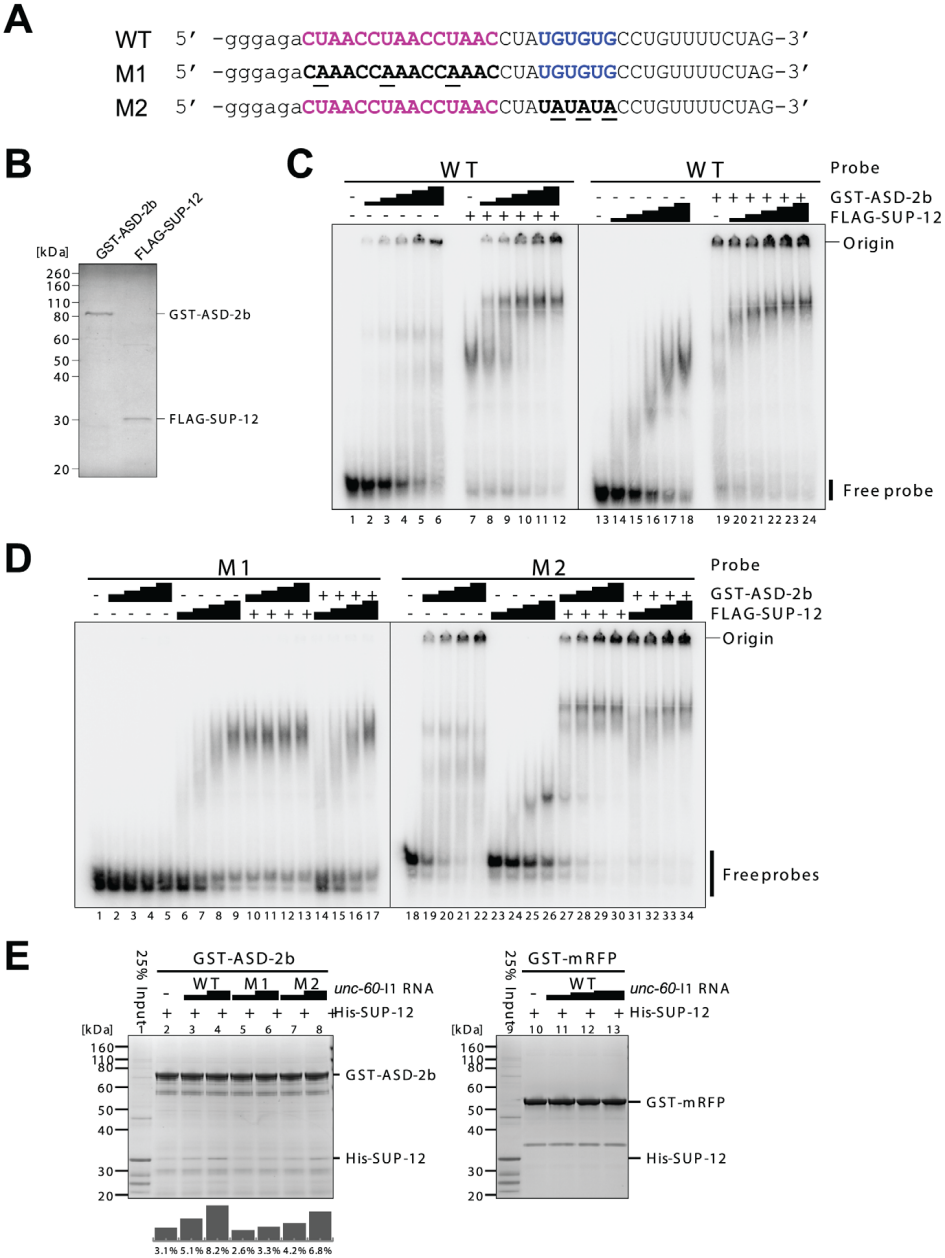
ASD-2 and SUP-12 cooperatively bind to *unc-60* intron 1A *in vitro* via direct and specific binding to CUAAC repeats and UGUGUG stretch, respectively. (*A*) Sequences of radiolabelled WT, M1 and M2 probes used in EMSAs. The sequences are illustrated as in Figure 3B, bottom panel. Lowercase indicates residues derived from T7 promoter. (*B*) SDS-PAGE and CBB staining of recombinant GST-fused full-length ASD-2b (GST-ASD-2b) and FLAG-tagged full-length SUP-12 (FLAG-SUP-12) proteins used in (*C*) and (*D*). (*C* and *D*) EMSAs using WT (*C*), M1 (*D*, lanes 1–17) and M2 (*D*, lanes 18–34) probes with 2-fold dilution series of GST-ASD-2b or FLAG-SUP-12 protein alone or in combination. (+) indicates the maximal amounts of proteins used in the dilution series. (*E*) Pull-down experiments of recombinant His-tagged SUP-12 (His-SUP-12) protein with immobilized GST-fusion proteins. GST-ASD-2b (lanes 2–8) and GST-mRFP (lanes 10–13) were incubated with His-SUP-12, 25% of which was run in lanes 1 and 9, in the presence of various concentrations of wild-type (WT), M1 mutant and M2 mutant *unc-60* intron 1A (*unc-60*-I1A) RNAs. Bar graphs below the gel indicate the amounts of His-SUP-12 pulled down with GST-ASD-2b relative to the input (lane 1). A representative result from two repeated experiments is shown.

To confirm direct and specific binding of ASD-2 and SUP-12 to the *cis*-elements in *unc-60* intron 1A *in vitro*, we prepared radiolabelled RNA probes containing the intact sequence (WT) or those with mutations as in the mutant reporters (M1 and M2) (Figure 4A) and recombinant full-length ASD-2b and full-length SUP-12 proteins (Figure 4B) to perform electrophoretic mobility shift assays (EMSAs) (Figure 4C, 4D). Recombinant ASD-2b protein shifted the mobility of WT (Figure 4C, lanes 1–6) and M2 (Figure 4D, lanes 18–22) probes in a dose-dependent manner and not of M1 probe (Figure 4D, lanes 1–5), demonstrating direct and specific binding of ASD-2b to CUAAC repeats. On the other hand, recombinant SUP-12 protein shifted the mobility of WT (Figure 4C, lanes 13–18) and M1 (Figure 4D, lanes 6–9) probes to a similar extent in a dose-dependent manner and less efficiently of M2 probe (Figure 4D, lanes 23–26) to a less extent, demonstrating direct and specific binding of SUP-12 to UGUGUG stretch. The result also indicated that SUP-12 could bind to other site(s) in the probes with a lower affinity.

We next asked whether ASD-2b and SUP-12 cooperatively bind to *unc-60* intron 1A RNA. We analyzed supershifts of the mobility of the *unc-60* intron 1A probes by the combination of ASD-2b and SUP-12 in EMSAs (Figure 4C, 4D). ASD-2b efficiently supershifted the mobility of WT probe at lower concentrations in the presence of SUP-12 (Figure 4C, lanes 7–12) compared to ASD-2b alone (lanes 1–6). In the same way, SUP-12 supershifted the mobility of WT probe at lower concentrations in the presence of ASD-2b (lanes 19–24) compared to SUP-12 alone (lanes 13–18). These results indicated that ASD-2b and SUP-12 cooperatively form a stable ASD-2b/SUP-12/RNA ternary complex with *unc-60* intron 1A RNA. ASD-2b failed to supershift the mobility of M1 probe (Figure 4D, lanes 10–17), indicating that CUAAC repeats are essential for the ternary complex formation. SUP-12 less efficiently supershifted the mobility of M2 probe (Figure 4D, lanes 31–34) compared to WT probe (Figure 4C, lanes 21–24) in the presence of ASD-2b, indicating that UGUGUG stretch is involved in the ternary complex formation.

We finally asked whether ASD-2b and SUP-12 can preform a complex in the absence of *unc-60* intron 1A by pull-down experiments (Figure 4E). Glutathione-*S*-transferase (GST)-fused full-length ASD-2b protein pulled down a substantial amount of recombinant full-length SUP-12 protein in the absence of target RNAs (Figure 4E, lane 2) and wild-type (WT) *unc-60* intron 1A (*unc-60*-I1A) RNA enhanced the pull-down efficiency in a dose-dependent manner (lanes 3, 4). On the other hand, GST-fused monomeric RFP (mRFP) protein failed to pull down SUP-12 protein even in the presence of *unc-60*-I1A RNA (lanes 10–13), demonstrating specific interaction between ASD-2b and SUP-12. M1 and M2 mutant *unc-60*-I1A RNAs less effectively enhanced the interaction between ASD-2b and SUP-12 (lanes 5–8), consistent with their weaker or no ability to form a ternary complex (Figure 4D). We therefore concluded that ASD-2b and SUP-12 can weakly interact with each other and that *unc-60* intron 1A RNA promotes the formation of the stable ASD-2b/SUP-12/RNA ternary complex by providing juxtaposed CUAAC repeats and UGUGUG stretch that are specifically recognized by ASD-2b and SUP-12, respectively.

### Depletion of ASD-2 leads to substantial expression of endogenous UNC-60A in body wall muscles and restores motility of *unc-60B* mutant

**Figure 5:**
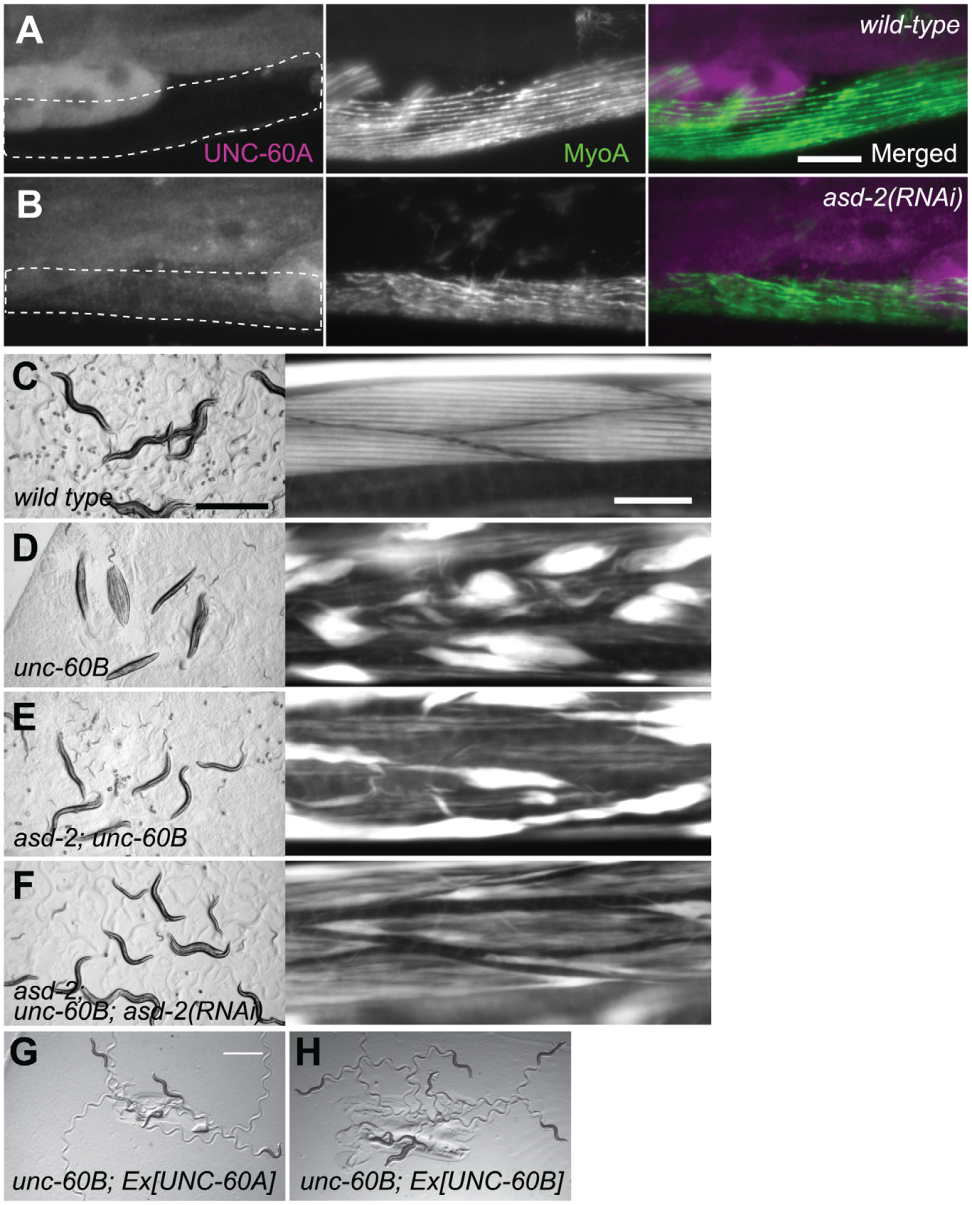
ASD-2 regulates alternative pre-mRNA processing of the endogenous *unc-60* gene in body wall muscles. (*A*, *B*) Immunofluorescence images of UNC-60A (left) and MyoA (middle) and merged images (right) of wild-type (*A*) and *asd-2(RNAi)* (*B*) worms. MyoA, a heavy chain of muscle-specific myosin, is a marker for body wall muscles (encircled with dotted lines). Scale bar, 20 µm. (*C*–*F*) Micrographs of worms on bacterial lawns (left) and actin filaments in body wall muscles stained with tetramethylrhodamine-phalloidin (right) of N2 (*C*), *unc-60 (su158)* (*D*), *asd-2 (yb1540); unc-60 (su158)* (*E*) and *asd-2 (yb1540); unc-60 (su158); asd-2 (RNAi)* (*F*). Scale bars, 1 mm in left panels and 20 µm in right panels. (*G*, *H*) Micrographs of *unc-60 (su158); ybEx2149 [myo-3::UNC-60A]* (*G*) and *unc-60 (su158); ybEx2148 [myo-3::UNC-60B]* (*H*) worms on bacterial lawns. Scale bar, 1 mm.

We examined whether ASD-2 regulates muscle-specific pre-mRNA processing of the endogenous *unc-60* gene. We have demonstrated that ASD-2 and SUP-12 cooperatively switch alternative processing of the *unc-60* reporter from UNC-60A-type to UNC-60B-type in body wall muscles. If this model can be applied to the endogenous *unc-60* gene, worms depleted of *asd-2* function should ectopically express UNC-60A in place of UNC-60B in body wall muscles. Indeed, RT-PCR analysis of the endogenous UNC-60 mRNAs revealed that relative amount of UNC-60B mRNA was decreased in *asd-2 (yb1540); asd-2 (RNAi)* worms (Figure S2). To further test the splicing change in body wall muscles, we investigated expression of UNC-60A protein by immunohistochemistry (Figure 5A, 5B). In wild-type worms, UNC-60A was undetectable in body wall muscles (Figure 5A, encircled) but was detected in other tissues (Figure 5A, left panel). Knockdown of the *asd-2* gene resulted in ectopic expression of UNC-60A in body wall muscles (Figure 5B, encircled), confirming that ASD-2 determines the processing patterns of the endogenous *unc-60* gene in body wall muscles.

Our previous work demonstrated that *sup-12* mutation strongly suppressed structural defects of body wall muscles and paralysis of UNC-60B-specific mutant, *unc-60B (su158)* [30]. The deletion allele *su158* lacks exons 3B and 4B (Figure 1A), and suppression of the phenotypes by *sup-12* mutation was likely due to ectopic expression of UNC-60A [30]. We therefore investigated whether knockdown of the *asd-2* gene also suppresses phenotypes of *unc-60B (su158)* mutant. Wild-type worms exhibited sinusoidal locomotion (Figure 5C, left panel), and actin filaments were organized in a striated pattern (Figure 5C, right panel). On the other hand, *unc-60B* (*su158)* worms were almost paralyzed (Figure 5D, left panel) with severe disorganization of actin filaments (Figure 5D, right panel). We found that *asd-2 (yb1540); unc-60B (su158)* double mutant slightly restored motility and actin filament organization (Figure 5E). Since *asd-2(RNAi)* worms showed a severer colour phenotype than *asd-2(yb1540)* allele (Figure 2C), we further knocked down remaining activity of *asd-2* by RNAi. As expected, *asd-2 (yb1540); unc-60B (su158); asd-2 (RNAi)* worms restored sinusoidal locomotion (Figure 5F, left panel) and actin filament organization was greatly improved (Figure 5F, right panel). We confirmed by immunohistochemistry that *asd-2 (yb1540)* mutation and/or *asd-2 (RNAi)* resulted in ectopic expression of UNC-60A in body wall muscles in the *unc-60B (su158)* background (Figure S3). Transgenic expression of UNC-60A (Figure 5G) as well as UNC-60B (Figure 5H) in body wall muscles restored sinusoidal locomotion of *unc-60B (su158)* mutant, indicating that UNC-60A can exert, at least in part, functions of muscle-specific UNC-60B isoform and that possible splicing change in other genes are not required for the phenotype suppression. These observations demonstrate that ASD-2 is a bona fide regulator of the muscle-specific pre-mRNA processing of the endogenous *unc-60* gene as well as SUP-12.

### SUP-12 represses excision of intron 1A from the endogenous *unc-60* transcript

**Figure 6:**
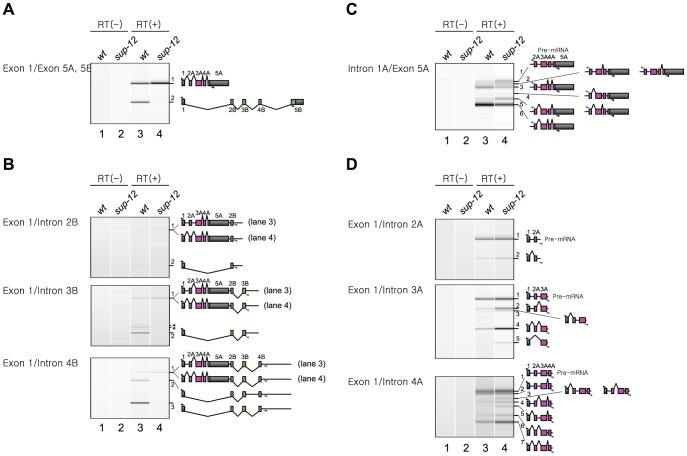
SUP-12 represses excision of intron 1A from the endogenous *unc-60* transcript. (*A*–*D*) RT-PCR analyses of mature mRNAs (*A*) and partially spliced RNAs (*B*–*D*) from the endogenous *unc-60* gene. Total RNAs from synchronized L1 larvae of N2 (lanes 1 and 3) and *sup-12 (yb1253)* mutant (lanes 2 and 4) were subjected to RT-PCR without (lanes 1 and 2) or with (lanes 3 and 4) reverse transcriptase (RT). Positions of the primers are indicated on the left. Each band is numbered in the order of size. Schematic structures of the RNAs are indicated on the right. Black and blue triangles indicate positions and directions of exonic and intronic primers, respectively. Asterisks denote artificially amplified fragments.

Finally, we analyzed splicing patterns of mature and partially spliced RNAs from the endogenous *unc-60* gene (Figure 6). For this experiment, we used wild-type and *sup-12 (yb1253)* worms because *asd-2 (yb1540)* mutation exhibited weaker effect on the *unc-60* reporter. In the wild type, mature UNC-60A and UNC-60B mRNAs were almost equally detected (Figure 6A, lane 3), while the latter was hardly detectable in *sup-12* mutant (lane 4), consistent with the result with the reporter (Figure 2H) and our previous study [30]. To analyze processing patterns of UNC-60B RNAs in body wall muscles, we amplified partially spliced RNAs carrying intron 2B, 3B or 4B with a forward primer in exon 1 and intronic reverse primers (Figure 6B). Partially spliced RNAs committed to UNC-60B, in which exon 1 was connected to exon 2B, were detected in the wild type (all panels, lane 3, bands 2 and 3) but were undetected in *sup-12* mutant (lane 4), consistent with the result shown in Figure 6A. These results indicated that SUP-12 is required for proper splicing between exon 1 and exon 2B in muscles. In *sup-12* mutant, all the introns, including intron 1A, were excised in the only detected RNAs (Figure 6B, all panels, lane 4, band 1), while in the wild type, intron 1A is retained in the longest detected RNAs (all panels, lane 3, band 1), indicating that SUP-12 represses excision of intron 1A.

We next analyzed partially spliced RNAs from the UNC-60A region (Figure 6C, 6D). Although the detected RNAs derived from this region were mixture of those in muscles and in non-muscle tissues, we assumed that differences in their relative amounts could be attributed to functions of SUP-12 in muscles. With a forward primer in intron 1A and a reverse primer in exon 5A (Figure 6C), we detected eight RNA species in *sup-12* mutant (lane 4, bands 1–6). These RNAs were all the theoretical intermediates in the UNC-60A processing. In the wild type (lane 3), two of the RNAs (bands 3 and 6) predominated, suggesting that SUP-12 represses their production. In these RNAs, intron 1A alone (band 6) or introns 1A and 2A were retained (band 3), supporting the idea that SUP-12 represses excision of intron 1A, and weakly of intron 2A, even after introns 3A and 4A are excised. We then analyzed the partially spliced RNAs with the forward primer in exon 1 and intronic reverse primers in introns 2A, 3A and 4A (Figure 6D). All the two (top panel, band 1–2), four (middle panel, bands 1–4) and eight (bottom panel, bands 1–7) theoretical intermediate RNA species were detected in *sup-12* mutant (lane 4), and relative amounts of the partially spliced RNAs to the pre-mRNAs (band 1) in the wild type (lane 3) and *sup-12* mutant (lane 4) were in good accordance with the idea that excision of introns 1A and 2A is facilitated in the absence of SUP-12. All these analyses of the partially spliced RNAs supported the model that SUP-12 represses excision of intron 1A to preserve exon 1 until exon 2B is transcribed in muscles.

## Discussion

**Figure 7:**
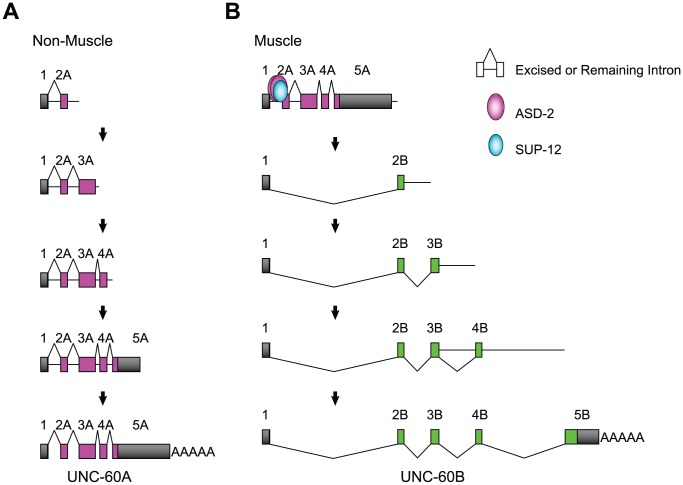
Schematic illustrations of the tissue-specific alternative processing of the *unc-60* pre-mRNA during the course of transcription. (*A*) A model of UNC-60A mRNA processing in non-muscle tissues. (*B*) A model of UNC-60B mRNA processing in muscles. See Discussion for detail.

In this study, we have provided genetic and biochemical analyses of the mechanisms for regulation of the muscle-specific alternative processing of the *unc-60* pre-mRNA. Figure 7 illustrates models of the pre-mRNA processing deduced from this study. In non-muscle tissues (Figure 7A), intron 1A and the other introns are excised during or after transcription and UNC-60A mRNA is generated. The order of intron removal is not strictly regulated as suggested by the presence of all the theoretical partially spliced RNAs (Figure 6C, 6D). In muscles (Figure 7B), ASD-2b and SUP-12 cooperatively bind to CUAAC repeats and UGUGUG stretch, respectively, in intron 1A to repress excision of intron 1A and weakly of intron 2A during transcription of the UNC-60A region. When UNC-60B-specific region is being transcribed, exon 1 is readily spliced to exon 2B, and introns 3B and 4B are also readily removed in the order of transcription (Figure 6B). Introns 3A and 4A are properly and rapidly excised during the UNC-60B processing (Figure 6C) likely due to their small sizes (53 nt and 60 nt, respectively). This may explain why exon 1 is not aberrantly spliced to exons 3A or 4A but is exclusively spliced to exon 2B to form UNC-60B mRNA. Regulation of tissue-specific alternative polyadenylation may also be involved in the fate-decision of the *unc-60* transcript, although the results demonstrated above did not provide conclusive evidence that ASD-2 and/or SUP-12 regulate muscle-specific repression of the polyadenylation site for UNC-60A mRNA.

We have demonstrated that ASD-2 and SUP-12 cooperatively represses the 3′-splice site and not the 5′-splice site of intron 1A. Although *C. elegans* does not have a recognizable branch point consensus or a polypyrimidine tract [42], a putative branch site for intron 1A is the A at position -19, between CUAAC repeats and UGUGUG stretch (Figure 3A). This A is the first A upstream from the 3′ splice site and is close to the positions where the putative branch site A is frequently found [43]. It is therefore reasonable to suggest that formation of ASD-2b/SUP-12/RNA ternary complex sterically hinders U2 snRNP auxiliary factor (U2AF) bound to the 3′-splice site from recruiting U2 snRNP to the branch site. The situation is quite similar to muscle-specific repression of *egl-15* exon 5B, where the Fox-1 family proteins and SUP-12 cooperatively bind to juxtaposed *cis*-elements overlapping a putative branch site [20], [38]. Recent microarray analyses of alternatively spliced exons in splicing factor mutants including *sup-12* identified many other splicing events affected by multiple splicing factors [44]. Combinatorial regulation by multiple splicing factors may be the common feature in tissue-specific alternative pre-mRNA processing in *C. elegans*.

ASD-2 ortholog in *Drosophila*, Held out wings (How) [45], [46], [47], and that in zebrafish, Quaking A (QkA) [48], are known to be required for muscle development or activity by mutant analyses. Vertebrate orthologs of SUP-12, known as SEB-4 or RBM24, are also expressed in muscle tissues and have recently been shown to be involved in myogenic differentiation by knockdown experiments [49], [50], [51], [52], [53]. However, the target events that these orthologs regulate in muscles remain almost unclear. Considering the highly conserved amino acid sequences and their expression patterns, it is likely that the orthologs of ASD-2 and SUP-12 regulate alternative pre-mRNA processing to produce muscle-specific protein isoforms in higher organisms.

In this study, we have presented a model of complex alternative pre-mRNA processing of a gene generating two almost distinct mRNAs. An important aspect of this study is the successful application of a dichromatic fluorescence reporter system to analyze the complex alternative pre-mRNA processing. The asymmetric pair of fluorescence reporter minigenes utilized in this study offers an alternative option for visualizing complex processing patterns besides symmetric pairs of minigenes applied to mutually exclusive exons and cassette exons [32], [33]. Another example of evolutionarily conserved genes with a structure similar to the *unc-60* gene is the cholinergic gene locus; genes encoding choline acetyltransferase (ChAT) and vesicular acetylcholine transporter (VAChT) share the common first exon, and the other exon(s) for VAChT reside in the first intron of the ChAT gene in mammals [54], *Drosophila* [55] and *C. elegans* [56]. The regulation mechanisms presented here would provide insight into the regulation of this kind of genes.

We demonstrated that ectopically expressed UNC-60A can compensate for the function of UNC-60B in sarcomeric actin organization in body wall muscles of *unc-60B* mutant. However, both UNC-60A and UNC-60B have characteristic actin-regulatory activities of ADF/cofilin *in vitro* with some quantitative differences [27], [28], [29]; UNC-60A has strong actin-monomer sequestering and only weak actin-filament severing activities, while UNC-60B has no actin-monomer sequestering and strong actin-filament severing activities. Although UNC-60A can compensate for the function of UNC-60B in body wall muscles, sarcomeric actin filaments in UNC-60A-complemented *unc-60B* mutant muscles still exhibit minor disorganization (unpublished data), suggesting that UNC-60B is a more suitable isoform. On the other hand, UNC-60B cannot compensate for the function of UNC-60A in the gonadal myoepithelial sheath [29]. This work and our previous works demonstrated that UNC-60A and UNC-60B are specifically adapted for functions in non-muscle and muscle cells, respectively, emphasizing that precise expression of appropriate ADF/cofilin isoforms, unravelled in this study, is important for development of tissue-specific actin-cytoskeletal structures [26], [29].

## Materials and Methods

### Plasmid construction

To construct the *unc-60E1-E2A-RFP* and *unc-60E1-E3B-GFP* cassettes, *unc-60* genomic fragments spanning from exon 1 through 2A and exon 1 through 3B, respectively, were amplified from N2 genomic DNA and cloned into Gateway Entry vectors (Invitrogen) carrying either mRFP1 [57] or EGFP (Clontech) cDNA by using In-Fusion system (BD Biosciences). *M1* and *M2* mutations were introduced by mutagenesis with Quickchange II (Stratagene). Expression vectors were constructed by homologous recombination between the Entry vectors and Destination vectors [31], [33] with LR Clonase II (Invitrogen). Sequences of the primers used in plasmid construction are available in Table S1.

### Worm culture and microscopy

Worms were cultured following standard methods. Transgenic lines were prepared essentially as described [33] using *lin-15 (n765)* as a host or pmyo-2-mRFP as a marker. Integrant lines were generated by ultraviolet light irradiation as described previously [33], [58]. Images of fluorescence reporter worms were captured using a fluorescence stereoscope (MZ16FA, Leica) with a dual and-pass filter GFP/DsRed equipped with a colour, cooled CCD camera (DP71, Olympus) or a confocal microscope (Fluoview FV500, Olympus) and processed with Metamorph (Molecular Devices) or Photoshop (Adobe).

### RNA interference

RNAi experiments by feeding were performed essentially as described [59]. Briefly, L4 hermaphrodites were transferred to agar plates seeded with bacteria expressing dsRNAs of target genes and their progeny were scored for colour and behavioural phenotypes or used for staining. For RNAi experiment by micro-injection, sense and anti-sense *asd-2* RNAs were prepared as described preciously [32] and were annealed at room temperature and 1–5 µg/µl dsRNA was injected into the gonad of young adult hermaphrodites. Injected worms were cultured at 20°C and the colour phenotype of their progeny was evaluated.

### RT–PCR

Total RNAs were extracted from worms by using RNeasy Mini kit (Qiagen) and DNase I (Promega). RNAs (300–500 ng) were reverse transcribed using random hexamers and Superscript II (Invitrogen) according to manufacturer's protocol. PCR was performed essentially as described previously [31], [33]. For amplification of partially spliced RNAs, total RNAs were reverse transcribed with PrimeScript II and random hexamers (Takara), and amplified with BIOTAQ (Bioline) and analyzed by using BioAnalyzer (Agilent). Sequences of the RT-PCR products were confirmed either by direct sequencing or by cloning and sequencing. Sequences of the primers used in the RT-PCR assays are available in Table S2.

### Recombinant proteins

Denatured His-tagged full-length ASD-2b for immunization was purified from denatured bacterial lysate by using Ni-NTA agarose (QIAGEN). Cold-shock inducible expression vectors for His-GST-fused full-length ASD-2b and mRFP1 and FLAG-tagged full-length SUP-12 were constructed by using Destination vectors pDEST-Cold-GST and pDEST-Cold-FLAG (H.K.), respectively. GST-ASD-2b and FLAG-SUP-12 were purified by using Glutathione Sepharose 4B (GE Healthcare) and Anti-FLAG M2 Magnetic Beads (Sigma), respectively, and dialyzed against RNA binding buffer (see below). Purified proteins were separated by standard SDS-PAGE and stained with SimplyBlue SafeStain (Invitrogen).

### Antibody production and Western blotting

Rabbit polyclonal anti-ASD-2b antiserum was generated with denatured recombinant His-ASD-2b protein by Operon Biotechnologies (Tokyo, Japan). IgG fraction (TD0135-02) was prepared from the antiserum by Medical & Biological Laboratories (Nagoya, Japan). Worm lysates were extracted from synchronized L1 larvae, separated by neutral polyacrylamide gel electrophoresis (NuPAGE, Invitrogen) and transferred to nitrocellulose membrane (Protran BA85, Whatman). Western blotting was performed with 15 µg/ml anti-ASD-2b (TD0135-02) or 1∶40,000-diluted anti-actin monoclonal antibody (Ab-1, Calbiochem) and 1∶1,000-diluted HRP-conjugated anti-rabbit IgG antibody (Pierce) or 1∶10,000-diluted HRP-conjugated anti-mouse IgM antibody (Calbiochem). Chemiluminescence signals (West Dura, Thermo) were detected by using LAS4000 (GE Healthcare).

### Immunohistochemistry

For staining with anti-ASD-2b, mixed stages of N2 and *asd-2 (yb1540)* worms were fixed with Bouin's fixative (15∶5∶1 mixture of saturated picric acid, formalin and acetic acid) supplemented with 25% methanol and 1.25% 2-mercaptoethanol for 60 min at room temperature, washed with phosphate-buffered saline (PBS) and permeabilized with 5% 2-mercaptoethanol and 1% Triton X-100 in PBS at 37°C for 30 hours. Fixed worms were treated with blocking buffer (0.5% skim milk and 0.5% bovine serum albumin (BSA) in PBS) for 2 hours at room temperature and stained with 6 µg/ml anti-ASD-2b (TD0135-02) as a primary antibody in blocking buffer for 24 hours at room temperature and then with 2 µg/ml Alexa488-conjugated goat anti-rabbit IgG (Invitrogen) as a secondary antibody together with 1 µg/ml Hoechst 33258 (Hoechst) in blocking buffer for 2 hours at room temperature. Fluorescence images were captured by using a compound microscope (DM6000B, Leica) equipped with a colour, cooled CCD camera (DFC310FX, Leica) or an inverted fluorescence microscope (Nikon TE2000) equipped with a monochrome CCD camera (SPOT RT, Diagnostic Instruments, Inc). Staining with anti-UNC-60A and anti-MyoA were performed as described previously [30]. Actin filaments were visualized by staining with tetramethylrhodamine-phalloidin as described previously [60].

### Electophoretic mobility shift assay (EMSA)

^32^P-labelled RNA probes were generated by *in vitro* transcription with [α^32^P] UTP (Perkin Elmer) and T7 RNA polymerase (Takara). Sequences of template oligo DNAs are available in Table S3. Gel-purified RNA probes alone or with increasing amounts of recombinant protein(s) were incubated in 25 µl of RNA binding buffer (20 mM HEPES-KOH (pH7.9), 150 mM KCl, 5% glycerol, 1% Triton X-100, 1 mM DTT and 0.1 mM PMSF) supplemented with 100 ng/µl *E. coli* tRNA and 50 ng/µl BSA for 30 min at 20°C. Each sample was separated on a non-denaturing 4% polyacrylamide gel and analyzed with a fluoro-imaging analyzer (FLA-3000G, Fuji Film).

### Pull-down

His-GST-fused recombinant full-length ASD-2b and mRFP1 proteins were immobilized on glutathione sepharose 4B beads (GE Healthcare) and incubated with His-SUP-12 in 100 µl of pull-down buffer (20 mM HEPES-KOH (pH7.9), 150 mM KCl, 1% Triton X-100, 1 mM DTT and 0.1 mM PMSF) supplemented with 100 ng/µl *E. coli* tRNA, 50 ng/µl BSA and 0, 30, 100, or 300 nM of *unc-60*-I1A RNAs (Operon Biotechnologies) for 30 min at 20°C. The sequences of the *unc-60*-I1A RNAs: *unc-60*-I1A-WT, 5′-UUUUUGCCUAACCUAACCUAACCUAUGUGUGCCUGUUUU-3′; *unc-60*-I1A-M1, 5′-UUUUUGCCAAACCAAACCAAACCUAUGUGUGCCUGUUUU-3′; *unc-60*-I1-M2, 5′-UUUUUGCCUAACCUAACCUAACCUAUAUAUACCUGUUUU-3′. Beads were washed four times with 1 ml pull-down buffer. Bound proteins were eluted with LDS sample buffer and separated by NuPAGE (Invitrogen). Gels were stained with SimplyBlue SafeStain (Invitrogen) and detected and analyzed by using LAS4000 (GE Healthcare).

## Supporting Information

Figure S1 RNAi knockdown of SUP-12-interacting proteins revealed ASD-2 as a candidate regulator of the *unc-60* reporter expression. Microphotographs of *ybIs1831* worms fed with bacterial clones expressing dsRNAs for indicated genes. Images in red channels are pseudo-coloured in magenta. Scale bar, 100 µm.
(PDF)

Figure S2 RT–PCR analysis of the endogenous *unc-60* mRNAs from synchronized L1 worms of N2 (lane 1), *asd-2 (yb1540)* (lane 2), *asd-2 (yb1540); ybIs1831; control (RNAi)* (lane 3) and *asd-2 (yb1540); ybIs1831; asd-2 (RNAi)* (lane 4). Splicing patterns of the mRNAs are schematically shown on the right. Triangles indicate positions and directions of the primers.
(PDF)

Figure S3 Immunofluorescence images of UNC-60A (left) and MyoA (middle) and merged images (right) of *unc-60 (su158)* (*A*), *unc-60 (su158); asd-2 (RNAi)* (*B*), *asd-2 (yb1540); unc-60 (su158)* (*C*) and *asd-2 (yb1540); unc-60 (su158); asd-2 (RNAi)* (*D*) worms. MyoA is a marker for body wall muscles (encircled with dotted lines in left panels). Scale bar, 20 µm.
(PDF)

Table S1 Sequences of primers used in plasmid construction.
(RTF)

Table S2 Sequences of primers used in RT–PCR assays.
(RTF)

Table S3 Sequences of oligo DNAs used in *in vitro* transcription.
(RTF)
