# Supplementary material for: Muscle-Specific Splicing Factors ASD-2 and SUP-12 Cooperatively Switch Alternative Pre-mRNA Processing Patterns of the ADF/Cofilin Gene in Caenorhabditis elegans
Source: PLoS Genet. 2012 Oct 11;8(10):e1002991. doi: 10.1371/journal.pgen.1002991 (PMC3469465; doi:10.1371/journal.pgen.1002991)
Supplement: Figure S1 — RNAi knockdown of SUP-12-interacting proteins revealed ASD-2 as a candidate regulator of the unc-60 reporter expression. Microphotographs of ybIs1831 worms fed with bacterial clones expressing dsRNAs for indicated genes. Images in red channels are pseudo-coloured in magenta. Scale bar, 100 µm. (PDF) [file pgen.1002991.s001.pdf]

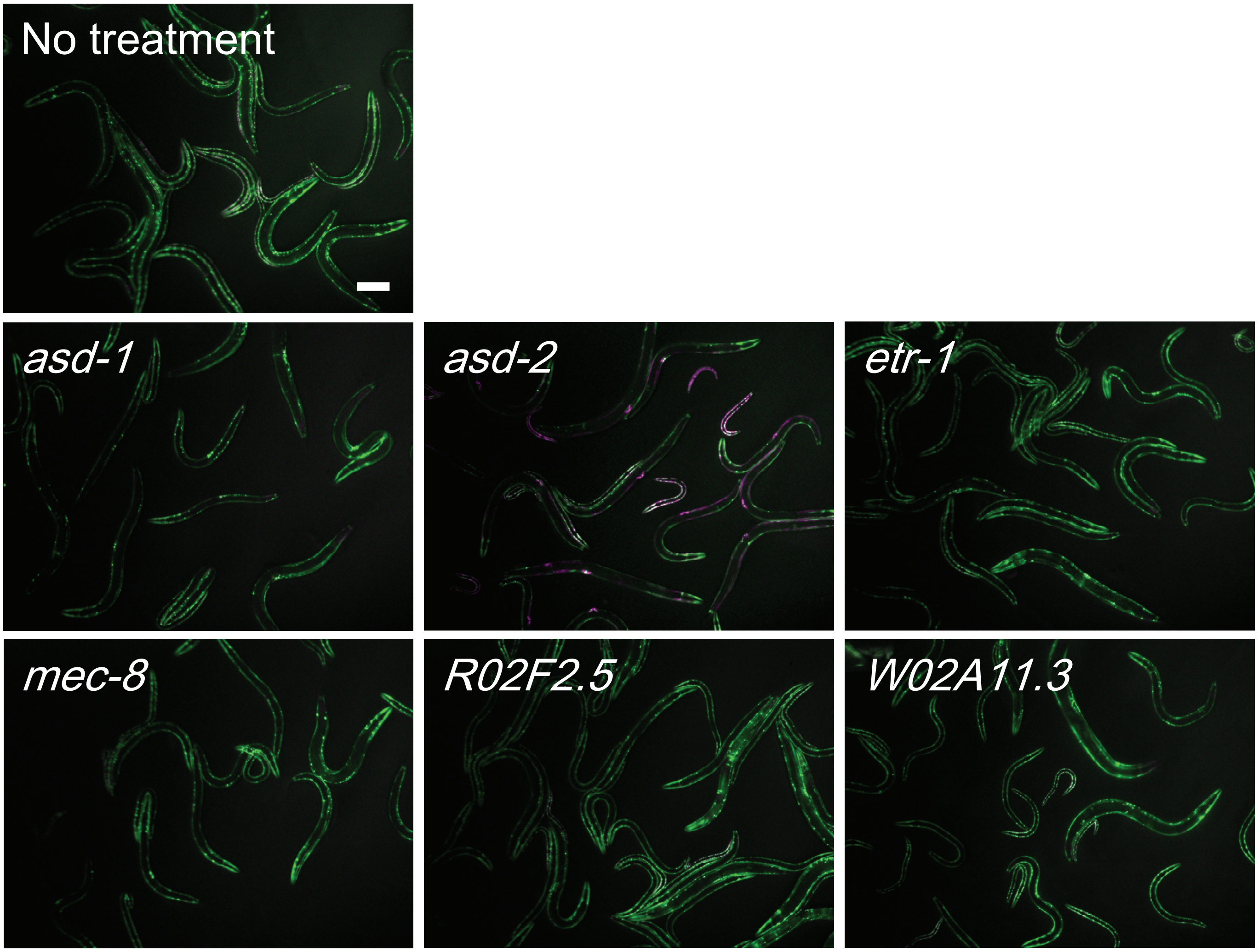

Figure S1. RNAi knockdown of SUP-12-interacting proteins revealed ASD-2 as a candidate regulator of the *unc-60* reporter expression. Microphotographs of *ybIs1831* worms fed with bacterial clones expressing dsRNAs for indicated genes. Images in red channels are pseudo-coloured in magenta. Scale bar, 100 µm.
