# Supplementary material for: Muscle-Specific Splicing Factors ASD-2 and SUP-12 Cooperatively Switch Alternative Pre-mRNA Processing Patterns of the ADF/Cofilin Gene in Caenorhabditis elegans
Source: PLoS Genet. 2012 Oct 11;8(10):e1002991. doi: 10.1371/journal.pgen.1002991 (PMC3469465; doi:10.1371/journal.pgen.1002991)
Supplement: Figure S2 — RT–PCR analysis of the endogenous unc-60 mRNAs from synchronized L1 worms of N2 (lane 1), asd-2 (yb1540) (lane 2), asd-2 (yb1540); ybIs1831; control (RNAi) (lane 3) and asd-2 (yb1540); ybIs1831; asd-2 (RNAi) (lane 4). Splicing patterns of the mRNAs are schematically shown on the right. Triangles indicate positions and directions of the primers. (PDF) [file pgen.1002991.s002.pdf]

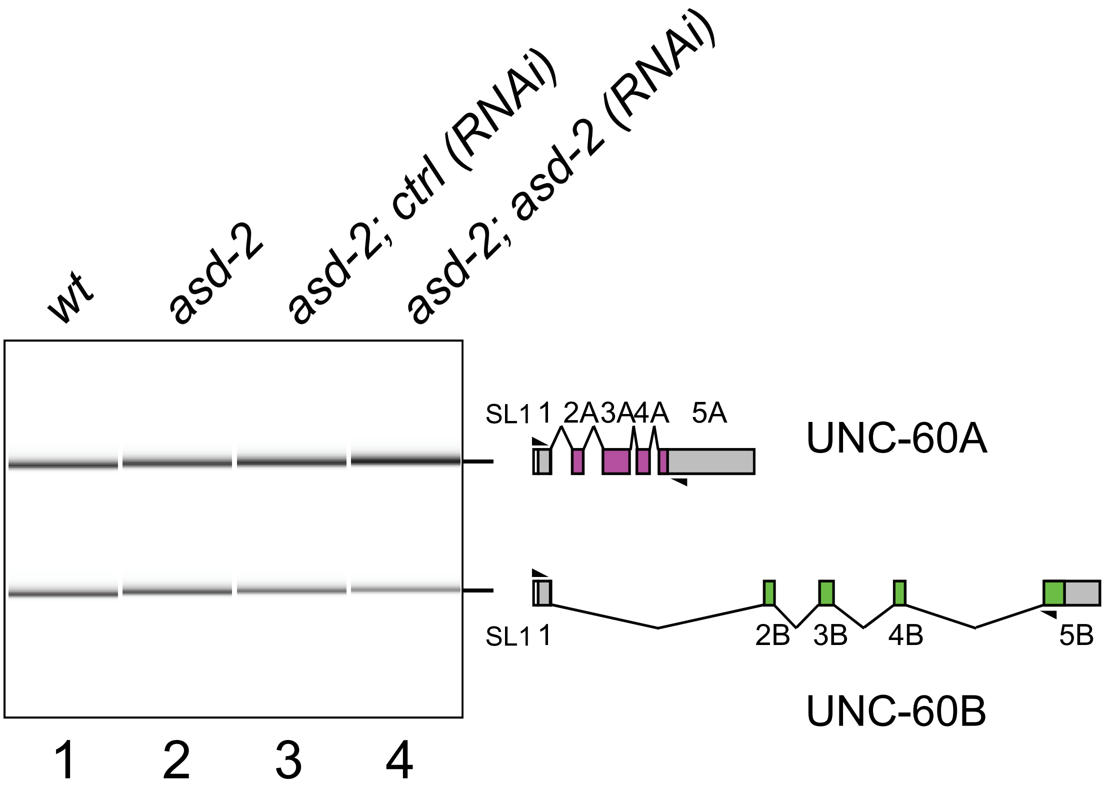

Figure S2. RT-PCR analysis of the endogenous *unc-60* mRNAs from synchronized L1 worms of N2 (lane 1), *asd-2 (yb1540)* (lane 2), *asd-2 (yb1540); ybIs1831; control (RNAi)* (lane 3) and *asd-2 (yb1540); ybIs1831; asd-2 (RNAi)* (lane 4). Splicing patterns of the mRNAs are schematically shown on the right. Triangles indicate positions and directions of the primers.
