# Supplementary material for: Muscle-Specific Splicing Factors ASD-2 and SUP-12 Cooperatively Switch Alternative Pre-mRNA Processing Patterns of the ADF/Cofilin Gene in Caenorhabditis elegans
Source: PLoS Genet. 2012 Oct 11;8(10):e1002991. doi: 10.1371/journal.pgen.1002991 (PMC3469465; doi:10.1371/journal.pgen.1002991)
Supplement: Figure S3 — Immunofluorescence images of UNC-60A (left) and MyoA (middle) and merged images (right) of unc-60 (su158) (A), unc-60 (su158); asd-2 (RNAi) (B), asd-2 (yb1540); unc-60 (su158) (C) and asd-2 (yb1540); unc-60 (su158); asd-2 (RNAi) (D) worms. MyoA is a marker for body wall muscles (encircled with dotted lines in left panels). Scale bar, 20 µm. (PDF) [file pgen.1002991.s003.pdf]

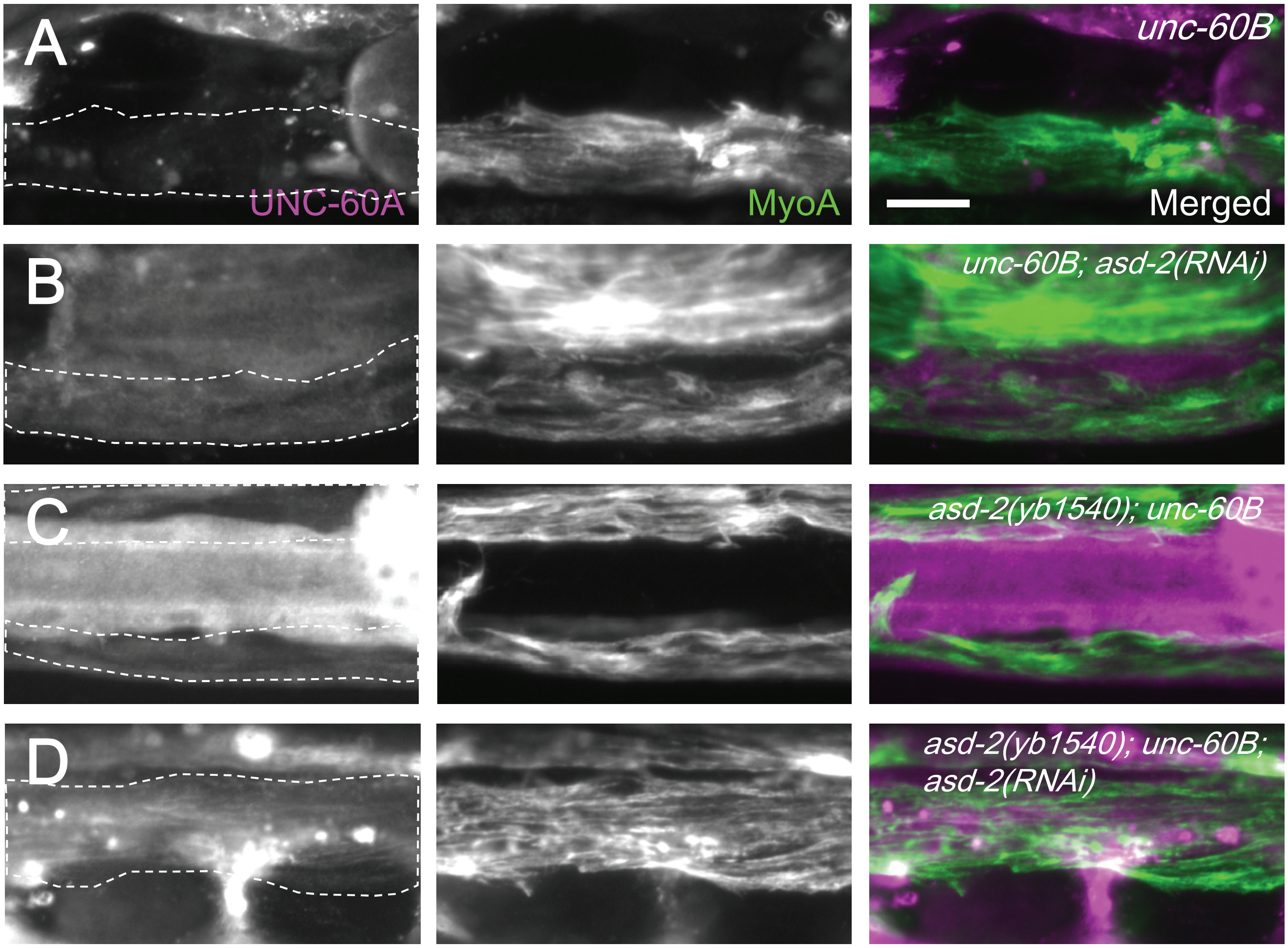

Figure S3. Immunofluorescence images of UNC-60A (left) and MyoA (middle) and merged images (right) of *unc-60 (su158)* (***A***), *unc-60 (su158); asd-2 (RNAi)* (***B***), *asd-2 (yb1540); unc-60 (su158)* (***C***) and *asd-2 (yb1540); unc-60 (su158); asd-2 (RNAi)* (***D***) worms. MyoA is a marker for body wall muscles (encircled with dotted lines in left panels). Scale bar, 20 μm.
