# Supplementary material for: Muscle-Specific Splicing Factors ASD-2 and SUP-12 Cooperatively Switch Alternative Pre-mRNA Processing Patterns of the ADF/Cofilin Gene in Caenorhabditis elegans
Source: PLoS Genet. 2012 Oct 11;8(10):e1002991. doi: 10.1371/journal.pgen.1002991 (PMC3469465; doi:10.1371/journal.pgen.1002991)
Supplement: Table S1 — Sequences of primers used in plasmid construction. (RTF) [file pgen.1002991.s004.rtf]

Table S1. Sequences of primers used in plasmid construction. 	
Primers used to amplify unc-60 genomic fragments	
Sequence	Constructed minigene cassettes	
5'-GGCTGCCGCTCTAGAGAAACTCAACTTGATTCTAT-3'	unc-60E1-E2A-RFP, unc-60E1-E3B-GFP	
5'-GCCATGGTGGGATCCAAAGATGTCTGCACATCTG-3'	unc-60E1-E2A-RFP	
5'-GCGACCGGTGGATCCCTGCACTCCTTTCCGTCCTC-3'	unc-60E1-E3B-GFP	
		
Primers used for mutagenesis	
Sequence	Constructed minigene cassettes	
5'-CTCTTCTTTTTGCCaAACCaAACCaAACCTATGTGTGCCTGTT-3'	unc-60E1-E2A-RFP-M1, unc-60E1-E3B-GFP-M1	
5'-AACAGGCACACATAGGTTtGGTTtGGTTtGGCAAAAAGAAGAG-3'	unc-60E1-E2A-RFP-M1, unc-60E1-E3B-GFP-M1	
5'-TAACCTAACCTATaTaTaCCTGTTTTCTAG-3'	unc-60E1-E2A-RFP-M2, unc-60E1-E3B-GFP-M2	
5'-CTAGAAAACAGGtAtAtATAGGTTAGGTTA-3'	unc-60E1-E2A-RFP-M2, unc-60E1-E3B-GFP-M2	
Lowercase indicates nucleotides different from wild type. 	
