# Supplementary material for: Muscle-Specific Splicing Factors ASD-2 and SUP-12 Cooperatively Switch Alternative Pre-mRNA Processing Patterns of the ADF/Cofilin Gene in Caenorhabditis elegans
Source: PLoS Genet. 2012 Oct 11;8(10):e1002991. doi: 10.1371/journal.pgen.1002991 (PMC3469465; doi:10.1371/journal.pgen.1002991)
Supplement: Table S2 — Sequences of primers used in RT–PCR assays. (RTF) [file pgen.1002991.s005.rtf]

Table S2. Sequences of primers used in RT-PCR assays. 	
Primers used to detect mRNAs derived from unc-60 reporter minigenes	
Name	Sequence	Target mRNAs	
attB1 forward	5'-GGGGACAAGTTTGTACAAAAAAGCAGGCT-3'	UNC-60A-RFP, UNC-60-I1A, UNC-60A-full, UNC-60B-GFP	
mRFPseqR	5'-GGAGCCGTACTGGAACTGAG-3'	UNC-60A-RFP, UNC-60-I1A	
unc-60#52	5'-CGACGACGACTTCGGAAGAGAC-3'	UNC-60A-full	
pEGFP#2	5'-TGTGGCCGTTTACGTCG-3'	UNC-60B-GFP	
Primers used to detect RNAs derived from the endogenous unc-60 gene	
Name	Sequence	Position & Direction	
UNC-60#5	5'-CCCAAGTTTGAGGAAACTCAAC-3'	SL1-Exon 1, Forward	
UNC-60#1	5'-GAAACTCAACTTGATTCTAT-3'	Exon 1, Forward	
unc-60#52	5'-CGACGACGACTTCGGAAGAGAC-3'	Exon 5A, Reverse	
UNC-60#16	5'-GACTGGGGCGTTGTCTGGGC-3'	Exon 5B, Reverse	
unc-60#53	5'-ACCTAACCTATGTGTGCCTG-3'	Intron 1A, Forward	
unc-60#66	5'-atagggaaatcaataatgttcatc-3'	Intron 2A, Reverse	
unc-60#56	5'-TCACCAATAGGATCAAGCGC-3'	Intron 3A, Reverse	
unc-60#68	5'-gtaaaaattatcgagatttctcacCTG-3'	Intron 4A, Reverse	
unc-60#70	5'-GACACAAGAGCTACAGTACTC-3'	Intron 2B, Reverse	
unc-60#60	5'-ggctcaggcctagtctcagg-3'	Intron 3B, Reverse	
unc-60#58	5'-TGGTTAAGAACGTGCTGACG-3'	Intron 4B, Reverse	
