# Supplementary material for: Muscle-Specific Splicing Factors ASD-2 and SUP-12 Cooperatively Switch Alternative Pre-mRNA Processing Patterns of the ADF/Cofilin Gene in Caenorhabditis elegans
Source: PLoS Genet. 2012 Oct 11;8(10):e1002991. doi: 10.1371/journal.pgen.1002991 (PMC3469465; doi:10.1371/journal.pgen.1002991)
Supplement: Table S3 — Sequences of oligo DNAs used in in vitro transcription. (RTF) [file pgen.1002991.s006.rtf]

Table S3. Sequences of oligo DNAs used in in vitro transcription. 	
Oligo DNAs used in preparing radiolabelled RNA probes for EMSA	
Name	Sequence	Probes	
T7p(+)	5'- TAATACGACTCACTATAGGGAGA-3'	All	
unc-60#T7_WT	5'-CTAGAAAACAGGCACACATAGGTTAGGTTAGGTTAGTCTCCCTATAGTGAGTCGTATTA-3'	WT	
unc-60#T7_M1	5'-CTAGAAAACAGGCACACATAGGTTtGGTTtGGTTtGTCTCCCTATAGTGAGTCGTATTA-3'	M1	
unc-60#T7_M2	5'-CTAGAAAACAGGtAtAtATAGGTTAGGTTAGGTTAGTCTCCCTATAGTGAGTCGTATTA-3'	M2	
Lowercase indicates nucleotides different from wild type. 		
